# *In Vitro* Antiplasmodium and Chloroquine Resistance Reversal Effects of Andrographolide

**DOI:** 10.1155/2019/7967980

**Published:** 2019-12-13

**Authors:** Zaid O. Ibraheem, Roslaini Abd Majid, Hasidah Mohd Sidek, Sabariah Md Noor, Mun Fei Yam, Mohammad Faruq Abd Rachman Isnadi, Rusliza Basir

**Affiliations:** ^1^Pharmacology and Toxicology Unit, Department of Pharmacy, Al Rafidain University College, Al Mustansyria, Baghdad, Iraq; ^2^Pharmacology Unit, Department of Human Anatomy, Faculty of Medicine and Health Sciences, Universiti Putra Malaysia, 43400 Serdang, Selangor, Malaysia; ^3^Department of Medical Microbiology and Parasitology, Faculty of Medicine and Health Sciences, Universiti Putra Malaysia, 43400 Serdang, Selangor, Malaysia; ^4^School of Bioscience and Biotechnology, Faculty of Science and Technology, Universiti Kebangsaan Malaysia, 43600 UKM Bangi, Selangor, Malaysia; ^5^Department of Hemeatology, Faculty of Medicine and Health Sciences, Universiti Putra Malaysia, 43400 Serdang, Selangor, Malaysia; ^6^Discipline of Pharmacology, School of Pharmaceutical Sciences, Universiti Sains Malaysia, 11800 Gelugor, Pulau Pinang, Malaysia

## Abstract

The emergence of drug-resistant strains of *Plasmodium falciparum* is the worst catastrophe that has ever confronted the dedicated efforts to eradicate malaria. This urged for searching other alternatives or sensitizers that reverse chloroquine resistance. In this experiment, the potential of andrographolide to inhibit plasmodial growth and reverse CQ resistance was tested *in vitro* using the SYBRE green-1-based drug sensitivity assay and isobologram technique, respectively. Its safety level toward mammalian cells was screened as well against Vero cells and RBCs using MTT-based drug sensitivity and RBC hemolysis assays, respectively. Its effect against hemozoin formation was screened using *β*-hematin formation and heme fractionation assays. Its molecular characters were determined using the conventional tests for the antioxidant effect measurement and the in silico molecular characterization using the online free chemi-informatic Molinspiration software. Results showed that andrographolide has a moderate antiplasmodium effect that does not entitle it to be a substituent for chloroquine. Furthermore, andrographolide ameliorated the sensitivity of the parasite to chloroquine. Besides, it showed an indirect inhibitory effect against hemozoin formation within the parasite and augmented the chloroquine-induced inhibition of hemozoin formation. The study suggests that its chloroquine resistance reversal effect may be due to inhibition of chloroquine accumulation or due to its impact on the biological activity of the parasite. Overall, this *in vitro* study is a clue for the reliability of andrographolide to be added with chloroquine for reversal of chloroquine resistance and tolerance, but further *in vivo* studies are recommended to confirm this notion. In spite of its prominent and safe *in vitro* and *in vivo* growth inhibitory effect and its *in vitro* chloroquine resistance reversing effect, it is inapplicable to implement it in malaria chemotherapy to substitute chloroquine or to reverse its resistance.

## 1. Introduction

Andrographolide is an interesting natural pharmacophore with lots of medicinal properties. It is isolated from an annual herbaceous plant called *Andrographis paniculata* (family: Acanthaceae). It has anticancer, anti-inflammatory, antibacterial, antiretroviral, antiparasitic, antiangiogenic [1], antithrombotic [2], antiurothelial [3], cardio- and hepatoprotective [4, 5], and immune-modulatory effects [6].

The emergence of chloroquine resistance in different strains of *Plasmodium falciparum* is the worst catastrophe that has ever perplexed the dedicated efforts to eradicate malaria. As it is still the most pertinent antimalarial drug due to its relative safety, efficiency, and cost effectivity in comparison with other conventional antimalarial drugs [7]. But, unfortunately it started to lose its token as a potential antimalarial drug due to the emergence of resistant and tolerant strains of *Plasmodium falciparum* [8, 9]. Trials to chemosensitizers from the sanctuary of phytochemicals are parts of the efforts to achieve this target.

Different pharmaceutical products were tested for chloroquine resistance reversal in the chloroquine-resistant strains of *Plasmodium falciparum*. Some had succeeded in this regard, such as calcium channel blockers, antihistamines, antipsychotics, tricyclic antidepressants, antipyretics, and NSAIDs (nonsteroidal anti-inflammatory drugs) [10]. It is suggested that most of them acted through inhibition of the functional activity of *pf*crt, the main transporter channel protein involved in the regulation of chloroquine accumulation within the digestive vacuole [11]. Previously, it was found that the development of chloroquine resistance is closely associated with mutational changes in its structure [12]. Then, it was found that its function is affected by the biochemical changes within the surrounding milieu. For instance, the chloroquine-reversing effect of verapamil was attributed to its aptitude to bind to certain allosteric sites within the *pf*crt. Till now, a clear molecular elucidation for its claimed action has not been achieved yet [13].

The synergy can be achieved when two drugs with different mechanisms are coadministered. During the intraerythrocytic stage, Hb is degraded by a series of plasmodial proteases, such as cysteine and serine proteases, to release the essential amino acids and soluble heme as a toxic byproduct. Heme interacts with the surrounding environment resulting in the unleashing of more free radicals and increasing the oxidative stress. Furthermore, it interferes with the digestive vacuole membrane integrity resulting in cellular autodigestion and ultimately necrosis or apoptosis. The parasite can cope with this dilemma by converting heme into an innocuous crystal called hemozoin. Chloroquine prevents this through binding to heme and capping the newly formed hemozoin, resulting in the prevention of its further biocrystallization. Furthermore, the chloroquine-heme complex tends to unleash more free radicals as compared with heme [14, 15].

On the contrary, recently, it was found that CQ may produce its effect through destabilization of the digestive vacuole membrane (lysmotropic effect) [16], increasing its permeabilization and leakage of the digestive vacuole content into the cytosol, such as calcium and cysteine proteases like cathepsin [16]. Once they leak out, they compromise the integrity of the mitochondrial membrane resulting in its permeabilization and leakage of its content of Cyt C (cytochrome C) and ROS (reactive oxygen species). This triggers the sequential cascade of the caspase activity that mediates DNA fragmentation and progression of the apoptotic pathway [17, 18].

At low doses, CQ induces the apoptotic features at a basal level, while at higher doses (micromolar concentrations), a higher number of apoptotic cells with MOMP and caspase overactivation evolve. Meanwhile, at the physiological nanomolar concentration, CQ accumulates inside the digestive vacuole and starts interfering with hemozoin formation. It starts appearing in the cytosol when its concentration jumps to micromolar concentration due to the digestive vacuole membrane permeabilization [16, 19].

All in all, the addition of any drug that can directly or indirectly enhance the progression of the mentioned mechanisms may synergize chloroquine and reverse its resistance in the resistant strains of *Plasmodium falciparum*. Since phytochemicals are Janus molecules that they act on multiple intracellular targets, their coadministration with chloroquine may change the way through which chloroquine produces its antiplasmodium effect.

This study aimed at testing the *in vitro* chloroquine resistance reversing effect of andrographolide using a model of *Plasmodium falciparum* K1-infected human RBCs. Andrographolide was chosen as an antiplasmodium candidate and chemosensitizer for chloroquine based on the previous literature that highlighted its significance as a phytochemical with medicinal properties.

## 2. Materials and Methods

### 2.1. Materials and Chemicals

RPMI-1640 medium, AlbuMax II, was procured from Gibco BRL (Grand Island, NY, USA). Meanwhile, each of HEPES (4-(2-hydroxyethyl)-1-piperazineethanesulfonic acid), triton X-100, EDTA, saponin, sorbitol, hypoxanthine, (100x) phosphate-buffered saline (PBS), bovine serum albumin (BSA), DMSO (dimethysulphoxide), and chloroquine diphosphate were purchased from Sigma-Aldrich (St. Louis, MO, USA). Furthermore, gentamicin was purchased from (Jiangxi Dongxu Chemical Technology Co., Ltd.), and andrographolide was purchased from Indofine Biochemical Company Inc. (cat no.: A-003). On the contrary, hemin chloride was procured from IKNOW (IKNOW, certificate number: GMP, SGS, HALA, KOSHER).

The parasites (*Plasmodium falciparum* K1) were procured from the Institute of Medical Research, Kuala Lumpur, Malaysia. Meanwhile, human O^+^ blood was obtained from the main author, pelleted, and washed using a washing medium containing RPMI-1640, 25 mM HEPES (4-(2-hydroxyethyl)-1-piperazineethansulphonic acid) buffer (pH 7.4), 24 mM sodium bicarbonate, 11 mM glucose, and 50 *μ*g/L gentamicin.

### 2.2. Molecular Characters Assessment

#### 2.2.1. Antioxidant Activity Measurement

*(1) Hydrogen Peroxide Scavenging Assay*. Hydrogen peroxide scavenging activity of andrographolide was determined according to Ruch et al. [20] wherein different concentrations of the drug (1 nM–1 mM) were incubated for 10 min with 30 mM solution of hydrogen peroxide prepared in 200 mM PBS (pH = 7.4). Then, the absorbance at 230 nm was measured spectrophotometrically using a (Jasco V550) spectrophotometer and 1 cm quartz cell. Results were compared with the absorbance of the drug-free hydrogen peroxide solution (positive control) and with H_2_O_2_-free PBS (negative control). The hydrogen peroxide scavenging activity was calculated as follows:

(1) $$\begin{array}{c} \%\text{ of }{\text{H}}_{2}{\text{O}}_{2}\text{ scavenging activity}=\frac{\left(Ac-Ab\right)-\left(As-Ab\right)}{\left(Ac-Ab\right)}\times 100, \end{array}$$

where *Ac*, *Ab*, and *As* represent absorbance values of the positive control, blank (the negative control), and the sample, respectively.

*(2) Reducing Power Assay*. Reducing power of each test compound was tested according to Oyaizu [21, 22], where 1 ml of different concentrations of each of andrographolide (1 nM–1 mM) prepared in ethanol were mixed with 5 ml of a solution containing equal volumes of potassium ferricyanide [K_3_Fe(CN)_6_] and PBS (200 mM and pH 6.5). The mixture was incubated for 20 min at room temperature, and then 2.5 ml of 10% TCA (w/v) was added to the mixture, and the mixture was centrifuged at 3000 rpm for 10 min. Then, 3 ml of the supernatant was mixed with an equal volume of D/W and 600 *µ*l of 0.1% ferric chloride, and finally the absorbance was measured spectrophotometrically at 700 nm. The extent of the absorbance augmentation is directly proportional to the reducing power of the compound. Results are expressed as % of absorbance augmentation [21, 22].

(2) $$\begin{array}{c} \%\text{ of reducing activity}=\frac{As}{Ac}\times 10, \end{array}$$

where both *As* and *Ac* represent absorbance of each sample and control, respectively.

*(3) DPPH Radical Scavenging Assay*. The aptitude of each compound to scavenge DPPH radical was performed as per Hatano et al's study. In brief, 20 *µ*l of different concentrations of andrographolide (1 nM–1 mM), dissolved in ethanol, were mixed with 180 *µ*l of 60 *µ*M DPPH (prepared with ethanol as well) using a 96-well plate and incubated in dark at room temperature for 60 min, and then the absorbance was measured spectrophotometrically at 517 nm (Versamax™) to measure the DPPH scavenging activity [23].

#### 2.2.2. Calculation of Physiochemical Properties and Bioactivity Prediction

Both of the physiochemical properties and the predicted bioactivity parameters of andrographolide were determined using chemi-informatic software known as Molinspiration (http://www.molinspiration.com). It performs a fragment-based virtual screening of the following parameters, namely, cLOGP (logarithm of octanol/water partition coefficient), PSA (molecular polar surface area), nON (number of nonhydrogen atoms), nOHNH (number of hydrogen-donating bonds), and nrotb (number of rotatable bonds). Furthermore, the software gives predictive drug-likeness scores toward the following intracellular targets: GPCR, kinase, nuclear factors, ion channels, and protease enzyme.

### 2.3. Parasite Culturing, Maintenance, and Synchronization

#### 2.3.1. Parasite Culturing and Maintenance

An inoculum of the procured *Plasmodium falciparum* K1 was cultured in a suspension of O^+^ red blood cells and suspended in a complete malaria culture medium (cMCM). cMCM was prepared by adding 0.75 mM hypoxanthine and 0.5% albumax (Gibco BRL) along with the same content of the abovementioned washing medium. Both pH and hematocrit of the cell culture suspension were maintained at 7.4 and 2%, respectively. The cultured parasites were incubated at 37°C in a microaerophilic atmosphere containing 90% N_2_, 5% CO_2_, and 5% O_2_. After incubation, the parasitemia progression was checked on a daily basis through Giemsa-stained smear technique along with changing the culture medium [24, 25].

#### 2.3.2. Parasite Synchronization

The cultured parasites were synchronized as previously described by Lambros and Venderberge [26]. Parasite synchronization is one of the prerequisites steps before the commencement of the drug sensitivity assay. In brief, the pelleted, unsynchronized, parasitized red blood cells (PRBCs) were incubated with an equal volume of 5% (w/v) sorbitol solution for 10 minutes. Sorbitol was washed out thrice using RPMI-1640 washing medium (RPMI-1640, 25 mM HEPES, and 50 *µ*g/ml gentamicin). Sorbitol hydrolyzes all the trophozoite- or schizont-infested PRBCs leaving the uninfected or those infested with the ring stage intact. It is preferred to do such a step while the rings are predominant in the cell culture. Finally, the washed PRBCs are cultured in cMCM for further analysis [26].

### 2.4. Stock and Working Solution Preparation

Stock solutions of 100 mM of each of andrographolide and CQ were prepared using DMSO as a cosolvent at 1% for the former and PBS (phosphate buffer saline (pH 7.4)) for the latter. The stocks were diluted with D/W to prepare working solutions at a concentration range of 1 nM to 1 mM.

### 2.5. Malaria Drug Sensitivity Assay

Malaria drug sensitivity assay was performed as previously described by Vossen et al. [27] and Ibraheem et al. [28, 29]. In brief, a drug containing flat-bottomed 96-well microtiter plates, featured triplicate of two-fold serial dilution of each of chloroquine and andrographolide (1 nM–1 mM), were incubated for 48 hours at 37°C with a cell culture suspension of the PRBCs (parasitized red blood cells). Chloroquine was used as a comparator reference to evaluate the potency of andrographolide. Control wells, containing drug without RBCs, untreated RBCs (0% parasite growth), and untreated PRBCs (100% parasite growth) were allocated as well. The parasites were synchronized at the ring stage and maintained at final parasitemia and hematocrit levels of 1%. The final volume in each well was maintained at 100 *µ*l (50 *µ*l of drug dilution + 50 *µ*l of PRBCs suspension). Working solutions were prepared using cMCM at a final concentration of 1 *µ*M. Three plates were prepared for each drug. After incubation, the plates were freeze-thawed, and 100 *µ*l of SYBR green-I lysis buffer (20 mM Tris, 5 mM EDTA, 0.008% saponin, and 0.008% triton-X-100) was loaded to each well. Then, the plates were incubated at room temperature for 1 hour, and finally the fluorescence was measured twice after 15 seconds of plate agitation using Victor Plate reader (Perkin Elmer, Salem, MA) at an excitation/emission wavelength of 485/535 nm. The geometric mean of the first and second pass was used to exclude any measurement error [27–29].

#### 2.5.1. Determination of Growth Parameters

Both IC_50_ and IC_90_ (concentrations required to inhibit the growth of 50% and 90% of the parasite growth, respectively) for each test drug against *Plasmodium falciparum* K1 were determined using after extrapolation of the log- (dose-) response curve using GraphPad prism version 6.

### 2.6. Effect on RBC Stability

Different concentrations of each dug (1 nM–1 mM) were incubated with O +ve human RBCs in an incomplete culture medium iCM (RPMI-1640, 25 mM HEPES, and 20 *µ*g/ml gentamicin) at 37°C for 48 hours using a 24-well plate (1 ml/cell). After incubation, 500 *µ*l from each well was transferred to Eppendorf tubes after thorough mixing, centrifuged at 500 g for 5 min, and 200 *µ*l of the supernatants were loaded into a flat bottomed 96-well plate to measure the released hemoglobin at 540 nm (VersaMaxtm). Results were compared with both negative and positive controls wherein the RBCs were incubated with a drug-free media and a medium containing 1% Tween 20. Tween 100 was used to produce a positive control with 100% hemolysis. RBCs hemolysis at each concentration was calculated as follows.

(3) $$\begin{array}{c} \%\text{ of hemolysis}=\frac{\left(As-An\right)}{\left(Ap-An\right)}\times 100. \end{array}$$

### 2.7. Effect of Andrographolide on Vero Cells

Vero cells (ATCC) were incubated for 48 hours with different concentrations of andrographolide (1 nM–1 *µ*M) at 37°C 5% CO_2_, 5% O_2_, and 90% N_2_ in a culture medium containing RPMI-1640, 10% BSA (bovine serum albumin), and antibiotic (100 U/ml penicillin and 100 *µ*g/ml streptomycin). After incubation, MTT assay was performed as previously described [30]. In the end, a dose-response-curve was extrapolated, and IC_50_ of andrographolide against Vero cells was estimated using GraphPad Prism software version 6.

### 2.8. Selectivity Index

Cytotoxicity of andrographolide against the pathogen (*Plasmodium falciparum*) was compared with that against RBCs and Vero cells through the determination of its selectivity index (SI) (ratio of the drug IC_50_ against plasmodium to that against each of RBCs and Vero cells). It estimates the extent of the drug selectivity toward the parasite as compared with two types of mammalian cells: RBCs, where the plasmodium thrives and Vero cell, a representative of mammalian epithelial cells.

(4) $$\begin{array}{c} \text{SI}=\frac{{\text{IC}}_{50}\text{ of the drug against plasmodium falciparum}}{{\text{IC}}_{50}\text{ of the drug against mammalian cells RBCs or Vero cells}}. \end{array}$$

### 2.9. Drug Combination Assay and Isobologram Analysis

For drug combination assay, the method followed by Zaid et al. was adopted [28, 31]. In brief, working solutions of each drug were prepared from their stocks at concentrations equivalent to 16 times their IC_50_. The dilution was chosen such that IC_50_ of each fell in the fourth twofold serial dilution. Then, the two solutions were mixed at fixed ratios (10 : 0, 7 : 3, 5 : 5, 3 : 7, and 0 : 10; ratios of CQ/phytochemical). After that, 50 *µ*l of each combination was uploaded in triplicate in row H of the 96-well plate (cells H2–H11) and were serially diluted throughout a 96-flat-bottomed plate (rows G–B), while the peripheral wells of the plate were uploaded with 50 *µ*l of the drug, RBCs and PRBCs controls. Then, the mixture was incubated at the standard conditions for 48 hours and treated as in drug sensitivity assay to determine the parasite growth profile and estimate IC_50_ and IC_90_ of each combination separately. For each combination ratio, both FIC_50_ and FIC_90_ (fractional inhibitory concentration) were calculated from the ratio of the drug's IC_50_ or IC_90_ within the combination to those when the drug was incubated with the parasite alone.

(5) $$\begin{array}{c} {\text{FIC}}_{50}=\frac{{\text{IC}}_{50}\text{ of the drug in combination}}{{\text{IC}}_{50}\text{ of the drug when it is incubated alone}},\\ \text{total }{\text{FIC}}_{50}={\text{FIC}}_{50}\text{ CQ}+{\text{FIC}}_{50}\text{ andro,}\\ {\text{FIC}}_{90}=\frac{{\text{IC}}_{90}\text{ of the drug in combination}}{{\text{IC}}_{90}\text{ of the drug when it is incubated alone}},\\ \text{total }{\text{FIC}}_{90}={\text{FIC}}_{90}\text{ CQ}+{\text{FIC}}_{90}\text{ andro.} \end{array}$$

At the end, two isobologram curves were plotted: FIC_50_- and FIC_90_-based isobolograms. The former was done through extrapolation of the FIC_50_ values for each of andrographolide and chloroquine on the abscissa (*X*-axis) and ordinate (*Y*-axis), respectively. Similarly, the FIC_90_-based isobologram was extrapolated as the FIC_50_ one, but the FIC_90_s were extrapolated instead. The line that links the two drugs FICs is considered as line of additivity such that the interaction is considered as additive when the points fall on the line or total FIC is equal to 1, synergistic when they fall below the line of additivity and is considered as indifference or antagonistic if the points fall above that line respectively. It is only considered antagonism if the total FIC is >2 [28, 31].

### 2.10. Effect of Hemozoin Formation

#### 2.10.1. *β*-Hematin Formation Assay

*β*-Hematin formation assay is based on incubating the drug with hemin chloride and monitoring its effect on hemozoin polymerization. In brief, to 100 *µ*l of 8 mM hemin chloride, dissolved in DMSO, 100 *µ*l of different concentrations of each test compound (0.8–40 mM) was added in the Eppendorf tube (i.e., 0.1–5 molar equivalents of hemin solution). Control tubes were allocated and treated with D/W instead of the drug. After that, *β*-hematin formation was initiated by adding 200 *µ*l of 8 M acetate buffer (pH = 5). Then, the tubes were incubated at 37°C for 18 hours, centrifuged at 3000 g, and the pellet was collected. Then, it was dissolved in DMSO and recentrifuged again to get rid of the unreacted hematin, which suspends in the supernatant, leaving the second pellet which contains the pure *β*-hematin. In the end, 400 *µ*l of 0.1 N NaOH was added to each tube to dissolve *β*-hematin, and 100 *µ*l aliquots of the final solution were transferred to other tubes, diluted 4 times using NaOH in the same mentioned concentration, and the absorbance was measured using a visible-light spectrophotometer at 390 nm. Chloroquine in the same concentration range was used as a positive control and absorbance versus concentration curve of each test phytochemical was compared with that of chloroquine which is a model drug for heme capping and hindrance of *β*-hematin formation [32].

#### 2.10.2. Plasmodium Hemozoin Formation Assay (Pyridin-Hemochromgen Reaction Assay)

*(1) PRBCs Suspension Preparation*. Heme fractionation assay was done to determine the impact of each of chloroquine and andrographolide at their IC_50_ and IC_90_ concentrations as well as their combination mixtures at 3 : 7, 5 : 5, and 7 : 3 (chloroquine/andrographolide) on the relative amount of the iron-containing molecules: hemoglobin, heme, and hemozoin. The experiment was performed as previously described by [33]. Firstly, a parasite suspension (at 4% of rings and hematocrit 2%) was serially diluted with uninfected blood such that the Hct was maintained at the mentioned level. Then, the mixtures were incubated for 32 hours and used to harvest the PRBCs (200 *μ*l aliquots of the culture were loaded to Eppendorf tubes in quadruplet and exposed to the centrifugation to harvest the PRBCs).

Then, the harvested PRBCs were used to determine the relative amount of different iron fractions. The amount of iron in each fraction was determined through mixing 200 *μ*l of the fraction with the pyridine hemochrome reagent (100 *μ*l of 4% SDS, 2.5% pyridine, and pH 7.5) for 15 minutes to form pyridine (Fe III) complex whose absorbance is determined spectrophotometrically at 405 nm.

First, the parasites were freed from the PRBCs using saponine lysis [23] (the cells were exposed to the saponin lysis buffer (0.05% saponin)) and then to the centrifugation to pellet out the free parasites. The supernatant was used to determine the total parasite free hemoglobin, while the pellets were exposed to further heme fractionation. Then, the pelleted parasites were diluted with D/W up to 1 ml, kept in −80 to be processed for the determination of each of the heme and the hemozoin fractions.

*(2) Intraparasite Iron Fraction Detection*. Then, the previous pellet was thawed, washed thrice with 0.5 ml of PBS (pH = 7.4), and then subjected to the hypotonic lysis (100 *µ*l of water and 100 *µ*l of 0.02 M HEPES (pH 7.5) were added, and the samples were vortexed and sonicated for 10 minutes). Then, the sample was centrifuged to pellet out the hemozoin and taking out the heme-rich supernatant. The same pyridine hemochrome assay was used for the determination of the heme fraction. The hemozoin pellet was treated first with 100 *µ*l of NaOH for 5 minutes with sonication. Then, the solution was neutralized with 100 *µ*l of 0.02 M HEPES and 100 *µ*l of 0.3 M HCl before adding the hemochrome reagent.

The heme standard curve was derived by measuring the spectrum of different concentrations of hemin prepared by serial dilution using 0.3 M NaOH as a vehicle and mixed with hemochrome pyridine reagent. This helped in quantifying the amount of each heme fraction as compared with the total RBCs number and the parasitemia level. The parasitemia was detected microscopically through smearing 20 *μ*l of the final suspension as described in the Giemsa-stained thin blood smear technique.

The results were expressed as mean ± SEM of the amount of iron in femtogram (fg) per cell for each fraction, and the amount was plotted against the parasitemia to get the amount of Hb, heme, or Hz obtained from each fraction. The parasitemia at each drug concentration was deduced microscopically, and the amount of hemozoin produced with such parasitemia in the presence of the drug was compared with that when the drug was absent. Finally, bar graphs were constructed to show the amount of iron in each fraction at different parasitemia ranges for the control and when the drugs were added at their IC_50_ and IC_90_ values, and then the graph of the combinations were constructed [33].

## 3. Results

### 3.1. Antioxidant Activity of Andrographolide

The *in vitro* screening of the antioxidant activity of andrographolide revealed that it has a prominent antioxidant activity but at an extent less than that of reference comparator antioxidants, such as butylated hydroxyl toluene (BHT) or vitamin C. The results suggest also that its effect is stronger against free radicals evolved *n* lipid compartments as it produced more pronounced scavenging effect against DPPH, a lipid-soluble free radical (Table 1).

### 3.2. *In Silico* Molecular Characterization

The *in silico* physiochemical properties of andrographolide revealed that it does not violate the five rules of thumb of Lipinski rules so it is suitable to be suggested as a candidate drug (Table 2). Furthermore, the predictive tool of the software for the biological activity of andrographolide revealed the presence of a noticeable effect against some targets such as GPCR, nuclear receptors, and protease (Table 2).

### 3.3. Drug Sensitivity Assay (Effect against Plasmodium, Vero Cells, and RBCs)

SYBRE-green-1-based sensitivity assay was performed to determine IC_50_ of the growth inhibitory effects of each of chloroquine and andrographolide against *Plasmodium falciparum* K1. As per [34], according to the IC_50_ values, the compounds are categorized into groups of different potency levels (Table 3).

Unlike chloroquine whose potency is excellent (IC_50_ < 1 *μ*M) as described by Li and Vederas [34] criteria, andrographolide was categorized as a drug of good potency against *Plasmodium falciparum* 3D7 and K1(IC_50_ = (1.366 ± 0.032 and 1.202 ± 0.151) and (9.67 ± 0.75 and 8.01 ± 0.887) *μ*M, respectively) (Tables 3 and 4). This signifies that andrographolide cannot be set as an alternative to substitute CQ in malaria therapy. Furthermore, it produced a comparable effect against both *Plasmodium falciparum* 3D7 and K1 (Table 4). Meanwhile, the discrepancy is high in CQ that its IC_50_ against K1 strain was about more than 10 times higher than that against (265.3 ± 5.5 and 21.19 ± 1.8 nM, respectively) (Table 4). Chloroquine potency against the two strains is considered excellent as per Li et al.'s classification but clinically, the strain is considered as resistant only if its IC_50_ is > 100 *µ*M. By this, 3D7 is sensitive, while K1 is resistant to chloroquine.

**Figure 1:**
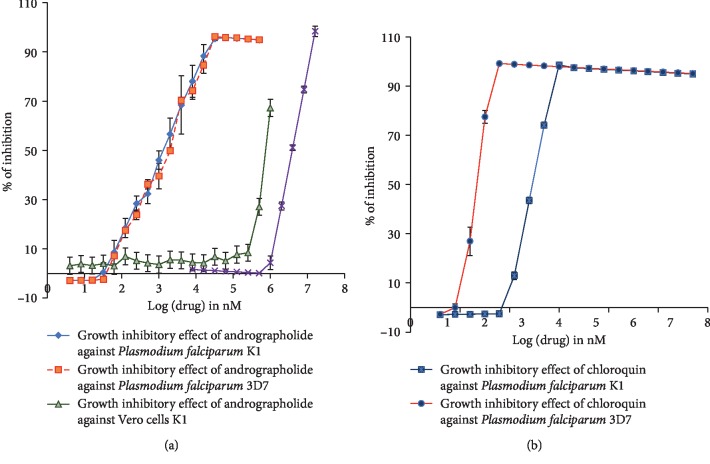
Antiplasmodium effect of each of andrographolide (a) and chloroquine (b) against *Plasmodium falciparum* 3D7 and K1. The chloroquine susceptible and resistant strains of *Plasmodium falciparum*. (a) also shows the dose-response curve for the cytotoxic effect of andrographolide against each of RBCs and Vero cells. Such effects for chloroquine were absent.

Vero (green monkey cells) and RBCs (red blood cells) were chosen as a model to determine the cytotoxic effect and selectivity to plasmodium of each test compound. According to previous studies, the compound is considered cytotoxic against mammalian cells only if its IC_50_ exceeds 30 *μ*g/ml. Table 4 shows the molar equivalent of each of chloroquine and andrographolide to achieve this threshold as well as their effect against RBCs and Vero cells. Chloroquine showed a very mild and unnoticeable cytotoxic effect against each of RBCs and Vero cells. Meanwhile, andrographolide showed a stronger effect. In summary, both drugs are considered as innocuous to human cells as there IC_50_ values for their cytotoxic effect against Vero cells were >1 mM (Figure 1, Table 4).

Selectivity to plasmodium as compared with RBCs and Vero cells (SI_RBCs_ and SI_vero_) were represented by the ratio of the IC_50_ of the cytotoxic effect of each test drug against RBCs or Vero cells to that against the parasite growth. It gives an indication of the ability of the compound to halt plasmodial growth without affecting the integrity of the mammalian cells. In this regard, both andrographolide and CQ showed considerable selectivity to plasmodium as they were devoid of a significant hemolytic effect against RBCs or toxic effect against Vero cells with SI_RBCs_ and SI_vero_ >1000 (Figure 1 and Table 4).

### 3.4. Isobologram Analysis

**Figure 2:**
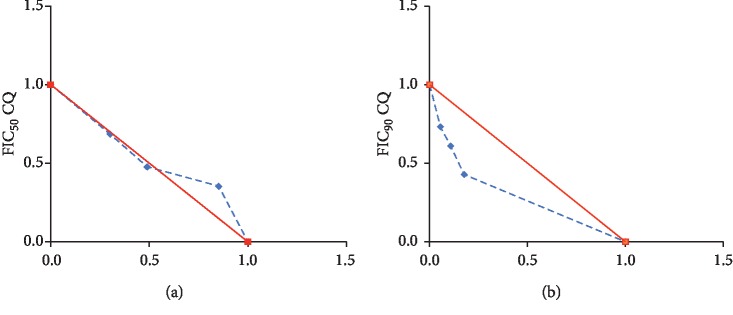
FIC_50_- and FIC_90_-based isobolograms for different combination mixtures of andrographolide and chloroquine at (10 : 0, 7 : 3, 5 : 5, 3 : 7, and 0 : 10 (chloroquine/andrographolide)). The red lines in the two graphs represent lines of additivity. Synergy is considered for the points located above the line of additivity while antagonism is considered for points located above that line.

Slight synergy was obtained between andrographolide and chloroquine as per results of IC_50_ and IC_90_ based isobolograms (Figure 2). As most of the points in the two curves fell slightly below the isobologram line of additivity.  Table 5 shows the extent at which the IC_50_ and IC_90_ of each compound had been affected in each combination.

Andrographolide interaction was more obvious in the IC_90_-based isobologram curve as compared with that in the IC_50_-based one. In the former, all the extrapolated points were located below the isobologram line indicating that andrographolide can reduce CQ tolerance at different mixing ratios. Meanwhile, at higher andrographolide/CQ ratio, effect of the combination on the IC_50_-based isobologram was additive as the FIC_50_-based extrapolation shows a point falling near the line of additivity. Interestingly, synergism was still there when andrographolide/CQ ratio was 3 : 7 or 5 : 5, as their correspondent FIC_90_-based extrapolation gives points below the line of additivity (Figure 2).

### 3.5. Effect on Hemozoin Formation

#### 3.5.1. Hematin Formation Assay

CQ showed a very high aptitude to inhibit heme polymerization and *β*-hematin formation with (IC_50_ = 53 *μ*M). Meanwhile, the ability of andrographolide to do so was relatively weak and produced this effect at 46 mM which is about one thousand times less than that of chloroquine. This indicates that the andrographolide does not have the due molecular characters that enable it to bind to hemozoin directly.

#### 3.5.2. Heme Fractionation Assay

**Figure 3:**
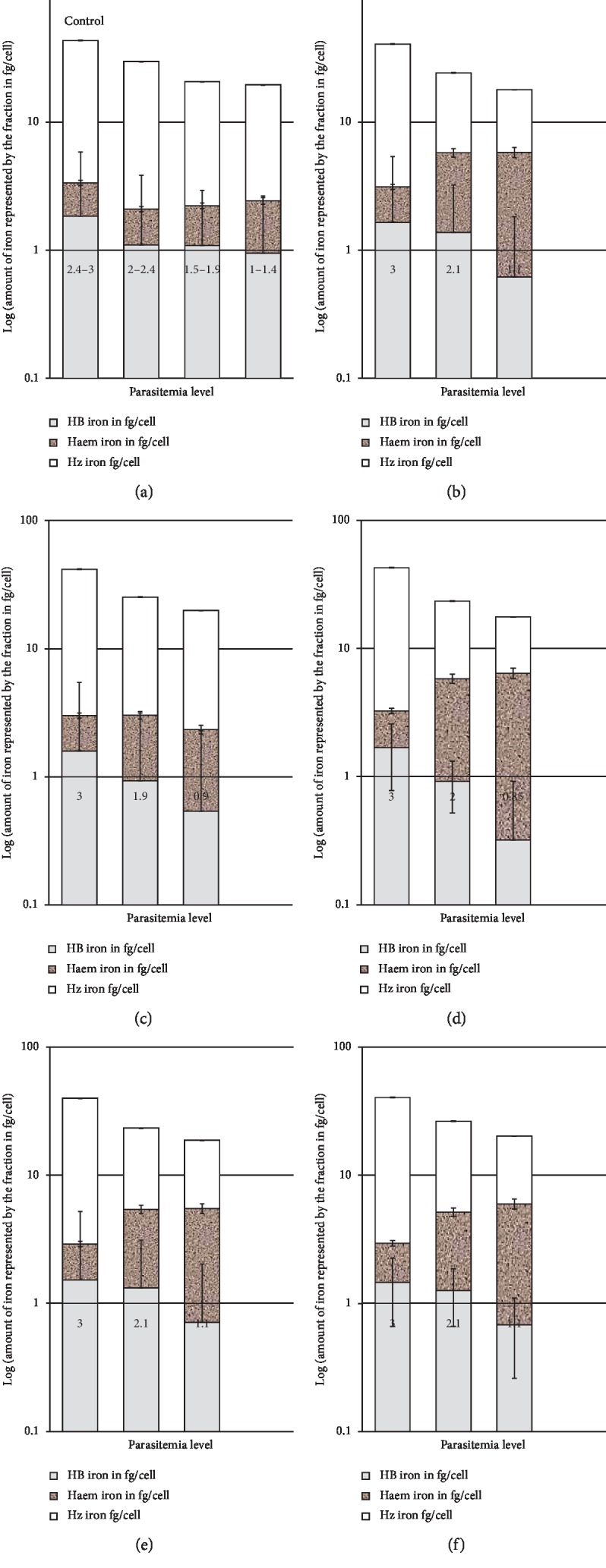
Amount of iron Hb, heme, and hemozoin iron produced in cultures *Plasmodium falciparum* K1 at different parasitemia in the control and untreated flasks (a) as well as in flasks treated with IC_50_ and IC_90_ concentration of each of chloroquine (b) and andrographolide (c). (d–f) show the amount of iron fractions (Hb, heme, or Hz) produced by the parasite after exposure to combinations of chloroquine and andrographolide at different combination levels (7 : 3, 5 : 5, and 3 : 7 (chloroquine/andrographolide)). The amount of iron in each fraction was estimated in fg of iron/cell. The results are expressed as log (amount of iron in each fraction in fg) in ordinate versus the parasitemia level in abscissa. *X* axis of the treated flask shows the parasitemia level after 24 h of exposure to the determined dose and the concentration of the drug (IC_50_ or IC_90_). For the combinations, both chloroquine and andrographolide were combined at their IC_50_ and IC_90_ at different ratios (7 : 3, 5 : 5, and 3 : 7 (chloroquine/andrographolide)). ^*∗*^, ^*∗∗*^, and ^*∗∗∗*^ signify statistically significant change as compared with that of the same parasitemia level of the positive control. While ^$^, ^$$^, and ^$$$^ signify a statistically significant difference as compared with the cohort flask of andrographolide-treated flasks.

The results showed that CQ produced a significant decline in the amount of released hemozoin and a significant increase in the amount of heme when it was used at concentrations equivalents to its IC_50_ (*P* < 0.01) and IC_90_ (*P* < 0.001). On the contrary, the andrographolide effect was weaker than that of chloroquine (*P* < 0.05). The decline in hemozoin formation and heme accumulation was noticed also after exposing the parasites to different combinations of chloroquine and andrographolide. The best combination was obtained when both drugs were mixed at 7 : 3 (CQ/andrographolide). The impact was slightly higher than that when the parasites were exposed to chloroquine only (Figure 3).

It is worth to note that unlike in the drug sensitivity assay, the exposure period of the parasite to the drugs was only 24 hours and during their active trophozoite stage. This made the lethality profile different from that of the drug sensitivity assay wherein the drug exposure is protracted to 48 hours.

## 4. Discussion

### 4.1. Introduction on Andrographolide

Andrographolide is a diterpenoid lactone belonging to the isoprenoid family of natural products. It is the main bioactive constituent of the stem and leaves of *Andrographis paniculata (Acanthaceae).* It is an interesting pharmacophore with plenty of medicinal and pharmacological effects [35, 36], such as antioxidant, antimicrobial, anti-inflammatory, and antitumor effects. Its growth inhibitory effect against microbial and tumor cells was ascribed to its impact on the proapoptotic pathway.

Previous studies highlighted the effect of andrographolide on different targets involved in the molecular machinery of the cell cycle, for instance, its inhibitory effect against P27 and cyclin-dependent kinase enzymes in tumor cells [37]. On the contrary, andrographolide revealed an immune-modulatory effect characterized by a prominent reduction in the efflux of cytokines from immune cells, such as IL2 and TNF-α production in immune cells [37, 38]. These facts were used as a base to choose andrographolide as a candidate chemosensitizer for chloroquine against *Plasmodium falciparum* K1, the chloroquine resistant strain of the parasite. Furthermore, its growth inhibitory effect against plasmodium was described previously in more than one study [39, 40].

### 4.2. Emergence of Chloroquine Resistance

Unfortunately, chloroquine started to lose its token as a reliable antimalarial due to the wide emergence of drug resistance among different strains of *Plasmodium falciparum*. This created a big obstacle in front of the efforts to eradicate the disease as in spite of this dilemma, it is still the safest and the most cost-effective drug amongst other antimalarial drugs. This fact urged the researchers to explore for chemosensitizers that can hinder the parasite resistance to chloroquine.

Not merely does the emergence of resistant phenotypes of *Plasmodium falciparum* limit CQ action. CQ pharmacokinetic characters constitute another obstacle against CQ success against malaria. The wide interindividual variation in the pharmacokinetic parameters of chloroquine has been highlighted previously [41, 42]. To solve this dilemma, reduction of the required dose to kill the parasite by adding chemosensitizers that reverse or ameliorate CQ resistance is considered as one of the proposed strategies.

### 4.3. *In Silico* Screening of Andrographolide Properties

The *in silico* physiochemical properties were determined using the online free chemi-informatic software known as Molinspiration (http://www.molinspiration.com). The software predicts the appropriateness of the compounds to be used as pharmaceutical drugs through the determination of its physiochemical properties and prediction of its biological activity against some common intracellular targets. This can be performed after introducing the structure of the compounds in a form of SMILES or SDF file. The main physiochemical properties that the software detects are *M* log *P* (Moriguchi log P algorithm), molecular weight, PSA (polar surface area in Å), number of hydrogen donors, number of hydrogen acceptor atoms, and number of Lipinski rule violations. The Lipinski rule of thumb states that to accept any compound as a reliable candidate to be used as a drug, it should not violate any of the following criteria. First, it should have a moderate lipophilicity (*M* log *P* should be < 5); second, its molecular mass should be <500 Dalton; third, it should not have more than 5 H donor groups and 10 H acceptor groups; and finally it should have a polar surface area (PSA) less than 140 Å. Interestingly, andrographolide fulfilled all of these criteria, and it showed moderate lipophilicity with a PSA of 87 A. Furthermore, its molecular mass and ubiquity of the H donor and acceptor groups were within the limit of the Lipinski rules.

On the contrary, the software was used to predict the intracellular biological activity of andrographolide by the determination of its drug likeliness score against some intracellular targets. This helps us in the interpretation of the results. Nevertheless, we should put in mind that results of this tool are not confirmative, but they are just auxiliary clues to interpret the results. Furthermore, the tool is designed for human models in which the targets are not 100% homologous with that of plasmodium. The main intracellular targets, against which the drug likeliness score was measured, were GPCR ligands (G protein-coupled-receptors), ion channels, kinases, nuclear receptor ligands, or proteases (http://www.molinspiration.com).

GPCR refers to a family of serpentine receptors that receive signals from the external environment to trigger an intracellular transduction signaling pathway and generate a cellular response. Previous studies pointed out to ubiquity of serpentine receptors homologs in plasmodium and revealed their involvement in recognition of the cell cycle-related transcription factors, regulation of the functional activity of merozoite specific protein (MSP), or eukaryotic initiation factor [43]. Andrographolide impact against GPCR was confirmed previously in some studies and to which its growth inhibitory and anti-inflammatory effects were attributed [44, 45].

Kinases are involved in transfer of phosphate from the high-energy phosphate-donating molecules to specific substrates. They are involved in several intracellular pathways, such as glycolysis, proteins, and lipids metabolisms, cell signaling cascade, cell cycle regulation, and secretion processes.

Plasmodium has plenty of kinase homologs, such as cyclin-dependent kinase, MAPK (mitogen-activated protein kinase) and carbohydrate kinases [46]. The effect of andrographolide on different kinases was highlighted in several studies, such as its impact on MAPK which is involved in the regulation of the immune response [47]. Nevertheless, the software gave a low drug likeliness score against different kinases.

Furthermore, the software predicted a good effect for andrographolide against the protease enzyme. This fact was seen previously against the HIV protease [48]. In plasmodium, there are two types of protease homologs, namely, serine and cysteine proteases. They are involved in Hb degradation which is a crucial pathway for plasmodium nutrition and induction of the plasmodium-related apoptotic pathway [49]. Its impact on this pathway may play a role in claiming its antiplasmodium effect [40].

### 4.4. Cytotoxic Effect of Andrographolide against Mammalian Cells

In our study, first, we assessed the cytotoxic effect of andrographolide against each of Vero cells (epithelial cells obtained from Kidneys of green monkeys) and RBCs. Andrographolide was found to be unhurtful to both cells as it produced its cytotoxic effect with IC_50_ >30 *μ*g/ml. Furthermore, its cytotoxicity against the mentioned cells was quite higher than their antiplasmodium making its selectivity toward plasmodium as compared with each of Vero and RBCs was high. Its impact on RBCs was attributed to the amphipathic nature that qualified it to accumulate in the double membrane layers of the RBCs membrane resulting in loss of its integrity and enhance RBCs lysis.

On the contrary, its effect against Vero cells can be ascribed to its aptitude to induce the apoptotic pathway and cell cycle arrest. The results showed that its cytotoxic effect against Vero cells was higher than that against RBCs, and this can be related to the presence of more targetable pathways within the former on which andrographolide may act. On the contrary, the results revealed that plasmodium had a higher sensitivity to andrographolide as compared with the mammalian cells. This can be attributed to the higher degree of development of the mammalian cells that make it more resistant to andrographolide or due to the ability of the drug to the target site present in plasmodium and absent in mammalian cells.

As a terpene derivative, it is suggested that andrographolide may have an impact against the specific isoprenoid biosynthesis pathway that is present in plasmodium and is absent in humans. Isoprenoids have myriads of biochemical effects within the cells ranging from regulation of the mitochondrial membrane potential to protein ubiquitination or isoprenylation [50]. In mammals, they are synthesized by the well-known mevalonate pathway, while in plasmodium, the biosynthesis takes place inside specific organelles called apicoplasts and follow a pathway called the nonmevalonate pathway (methylerythritol phosphate (MEP) pathway). MEP represents one of the attractive targets for the development of new antimalarial drugs. Previous studies have pointed out to the possible inhibitory role of different terpenes on this pathway [51].

Different drugs have different mechanisms to inhibit plasmodium growth. Most of them act on the digestive vacuole through interfering with hemoglobin breakdown or heme detoxification. The parasite breaks down hemoglobin into heme and globin to use the latter as the sole source of protein while heme is released as an obnoxious waste product. Heme is detoxified inside the digestive vacuole through a series of biocrystallization and biomineralization steps to produce an innocuous waste product called hemozoin. This step is crucial for survival of the parasite. This pathway may be targeted by drugs that inhibit hemoglobin breakdown resulting in cessation of amino acid supply to plasmodium or targeted by drugs that inhibit heme detoxification resulting in its accumulation within the plasmodial cytosol. Heme has a powerful prooxidant effect resulting in overaccumulation of free radicals and induction of cellular damage. Interference with heme detoxification is the main mechanism of chloroquine, the most widely used conventional antimalarial chemotherapy.

### 4.5. Chemosensitizing Effect of Andrographolide

In this study, we investigated the plausible synergy between CQ and andrographolide using the well-known isobologram technique. The technique had been used widely to deduce the mode of interaction between two different compounds [52]. Two isobologram curves were plotted: IC_50_-and IC_90_-based isobolograms, to investigate the potential of andrographolide to reverse CQ resistance and tolerance. Drug resistance can be deduced from IC_50_ values of drug sensitivity assays and according to Stephanie et al. [9], tolerant phenotypes have characteristically higher values of IC_90_. Resistance incurs when the parasite loses its response to a high dose of the drug [53], while tolerance is associated with protraction of the required period to attain complete eradication of the parasite [9]. Both problems encounter different strains of *Plasmodium falciparum* worldwide. They are predestined for raising the therapeutic dose and lengthening the treatment period.

Results of the isobologram study suggest the synergy between chloroquine and andrographolide. Nevertheless, the synergy was still weak as all the extrapolated points were located at positions slightly lower than the isobologram line and not all the interactions produced total fractional inhibitory concentrations (total FIC) are between 0.5 and 1. Strong synergy is obtained if the FIC_total_ is below 0.5, but the synergy is considered weak as it approaches one. Furthermore, the plot shows that the synergy according to the FIC_90_-based isobologram is higher than that of the FIC_50_ one. This suggests that andrographolide has a higher potential to inhibit tolerance to chloroquine rather than its impact on resistance. Previous studies have attributed drug tolerance to the ability of the parasite to recrudesce after giving the therapeutic dose to the drug [9].

Normally, drug tolerance is screened through extrapolating the parasite clearance curve [54]. Nevertheless, some resources highlighted the linkage of drugs IC_90_ to the parasite tolerance [55]. This indicates that andrographolide can shorten the time required for the chloroquine-induced complete eradication of the parasite.

Although it failed to completely reverse CQ resistance in *Plasmodium falciparum* K1, andrographolide showed a potential to ameliorate it as it changed the responsiveness of the parasite from the high resistance mode into the moderate one as CQ IC_50_ was dropped from 265 *µ*M to 106 *µ*M when the two drugs were mixed at 5 : 5 (andrographolide/CQ) ratio and to about 88 *µ*M when they are mixed at 7 : 3.

The emergence of chloroquine resistance in different strains of *Plasmodium falciparum* is the worst catastrophe that has ever perplexed the dedicated efforts to eradicate malaria. Different chemicals were tested as chemosensitizers to improve CQ effect against both CQ resistant and sensitive strains of *Plasmodium falciparum*. Some of them succeeded to be effective candidates to improve CQ sensitivity against the CQ resistant strains of *Plasmodium falciparum*. They belong to different categories of pharmaceutical drugs, such as calcium channel blockers, antihistamines, antipsychotics, and tricyclic antidepressants [10]. It is noteworthy that all of them did not improve chloroquine sensitivity against the sensitive strains. This raises a notion that they may affect the functional characters of *pf*crt, the main transporter channel protein involved in the regulation of chloroquine accumulation within the digestive vacuole [11].

Previous studies referred development of chloroquine resistance to mutational changes in *pf*crt, the transporter that regulates shuffling of chloroquine into the digestive vacuoles [12]. Later on, it was found that its function is affected by the biochemical changes within the surrounding milieu which may play a role in regulating its function. Till now, the studies failed to give a clear molecular elucidation for its claimed action although, previously, it was found that the chloroquine resistance reversing effect of verapamil was due to its direct binding to certain allosteric sites within the *pfcrt* transporter [13, 56].

Furthermore, the plausible impact of andrographolide against isoprenoid biosynthesis inside the apicoplast may have played a role in the phenomenon of synergy with chloroquine. Apicoplasts are organelles present in plasmodium and are thought to be relict organelles, remained after the evolution of the parasite. They are synthesized through a pathway different from that of the human cells. Isoprenoid biosynthesis occurs through the well-known mevalonate biosynthetic pathway in mammals, but in plasmodium, it is synthesized by a unique pathway called the nonmevalonate pathway (methyerthrytol pathway (MEP)) [50]. Isoprenoids are crucial for the functional activity of plasmodium cells. It is involved in isoprenylation and isoprenoid conjugation of different proteins. Furthermore, they are involved in the intracellular signaling transduction pathway. After Hb ingestion, Hb is packaged into small vesicles that translocate it from the cytostome toward the digestive vacuole. This translocation requires recognition of a series of isoprenylated proteins that their absence may hinder Hb deposition within the digestive vacuole [57]. This action may be conferred by agents that interfere with the apicoplast function that might be responsible for synergizing the antiplasmodium effect of chloroquine.

As mentioned earlier, chloroquine effect on hemozoin formation is not the mere mechanism through which chloroquine produces its effect. Its lysmotropic property that increases seeping of the digestive vacuole content into the cytosol is another mechanism. Ultimately, this pathway results in the induction of the cascade sequential pathway of the apoptotic-programmed cell death [58]. Previous studies found that andrographolide is a potent promoter of the apoptotic signal and acts through inhibiting the mitochondrial membrane potential resulting in outpouring of the mitochondrial content of cytochrome C, free radicals, and calcium into the cytosol and activation of the cascade of the caspase pathway that ends up in breaking of the cellular macromolecules of proteins and genetic materials into small fragments [59]. Further studies are recommended to investigate the contribution of the mentioned pathways in mediating the claimed impact of andrographolide.

Interestingly, when andrographolide ratio was increased as compared with CQ and reached 3 : 7 (CQ/andrographolide), the synergistic and additive impact was dwindled, and the effect was turned to be indifferent according to the FIC_50_-based isobologram. This may be ascribed to eruption of the antioxidant effect of andrographolide that entitles it to mop out the CQ-induced free radical generation. This action might have faded the proposed cytotoxic effect of andrographolide. Although it is well known that antioxidants are double sword weapons as they produce their protective effect at low concentrations and they turn into toxic prooxidants at higher concentration, this effect is seen only for highly potent antioxidants [60]. Its threshold to produce its prooxidant effect might be different in eukaryotic and prokaryotic cells. Results of the *in vitro* antioxidant activity assessment revealed that andrographolide has a good antioxidant potential but its effect did not match that of the standard antioxidants. This decreases the possibility of having the incidence of its prooxidant effect when it was combined at this ratio with CQ.

Recently, it was found that CQ may produce its effect through destabilization of the digestive vacuole membrane (lysmotropic effect) [16]. This effect highlighted a new avenue for antimalarial drug strategy development. Like any lysosomal membrane, the digestive vacuole membrane is lined by proteins known as LAMP (lysosomal-associated membrane protein) and LIMP (lysosomal integral membrane protein). They constitute 50% of the total membrane proteins and protect against various hydrolytic enzymes. As a lysmotropic drug, CQ binds to the membrane resulting in lysosomal membrane permeabilization and leakage of the lysosomal content into the cytosol, such as calcium, cytochrome C, and proteases like cathepsin and other cysteine proteases [16].

Once it leaks out, cathepsin attacks the outer mitochondrial membrane resulting in mitochondrial outer membrane permeabilization which triggers leakage of the mitochondrial content of Cyt C (cytochrome C) and ROS (reactive oxygen species) into the cytosol, progression of the sequential cascade of the caspase activity that mediates DNA fragmentation, and progression of the apoptotic pathway [17, 18].

At low doses, CQ induces the apoptotic features at a basal level, while at higher doses (micromolar concentrations), higher number of apoptotic cells with MOMP and caspase overactivation evolve. Meanwhile, at the physiological nanomolar concentration, CQ accumulates intravacuolarly and starts interfering with hemozoin formation. It starts appearing in the cytosol when its concentration jumps to micromolar concentration due to digestive vacuole membrane permeabilization [16].

Previous studies revealed that CQ induces a cidal effect as seen in results of Laura et al. [54], where the parasite viability dropped significantly in the subsequent cycles after CQ exposure. Checking the parasitemia after 48 hours (in the subsequent cycle) as what we did in the drug sensitivity assay, gives perception about the cidal effect as only alive cells from the previous cycle can progress into the next one. Static drugs do not affect the parasite viability but may protract the life cycle resulting in reduction of the parasites proliferation rate [54].

### 4.6. Effect on the Digestive Vacuoles

The impact of andrographolide on the digestive vacuole was investigated. First, its impact on heme polymerization was screened using *β*-hematin formation inhibition assay. The assay screens the ability of the compounds to mimic chloroquine action through capping heme moieties and halting its polymerization and hemozoin formation. Heme is a toxic byproduct of hemoglobin catabolism. Its obnoxious effect is hindered by its tendency to polymerize and convert to the innocuous hemozoin. Hemozoin formation requires the establishment of reciprocating iron oxygen bonds between the central iron of one of the ferroprotoporphyrin moieties and the carboxyl group of the other (*π*-*π* bonding) [61]. This bonding creates heme dimers that can stack together through establishing hydrogen bonds among the uncoordinated side chains. This process can be inhibited by drugs that can establish *π*-*π* bond with the ferroprotoporphyrin resulting in halting of the heme dimer and the subsequent hemozoin formation. CQ is a good example of such drugs as it contains hydroxyl moieties that entitle it to undergo this *π*-*π* bonding [61].

Results of the *β*-hematin formation inhibition assay shows that unlike chloroquine, andrographolide had a very weak effect against *β*-hematin formation. This excludes the presence of a direct inhibitory effect for andrographolide on hemozoin formation.

While performing the heme fractionation assay, both andrographolide and chloroquine reduced the amount of released hemozoin significantly and enhanced heme accumulation. The extent of this effect was higher for chloroquine as it was significantly different as compared with cohort flasks that contained similar parasitemia levels of the untreated flasks, with *P* < 0.001. Meanwhile, the decline in hemozoin formation was less after exposure to andrographolide, and the level was less than that of the untreated control with (*P* < 0.05).

Since andrographolide failed to produce a prominent direct inhibitory effect against heme polymerization, its inhibitory effect on the heme fractionation assay can be ascribed to its impact on the biological activity of the plasmodium resulting in less hemoglobin breakdown or less heme detoxification to hemozoin. It is noteworthy that hemoglobin digestion and uptake by the digestive vacuole is a sequence of processes that may be affected by drugs that interfere with the functional activity of plasmodial cells. Translocation of Hb into the digestive vacuoles requires a series of isoprenylated protiens. Previous studies revealed that terpenes like andrographolide may interfere with the pathways of isoprenoid biosynthesis and by this they can indirectly interfere with Hb translocation. As a terpene derivative, it is suggested that andrographolide interferes with the isoprenoid biosynthetic pathways in plasmodium. This may result in less protein prenylation and less influx of hemoglobin into the digestive vacuole. Further studies are recommended to confirm this notion [57].

## 5. Conclusion

Coadministration of andrographolide with chloroquine at ratios higher than 5 : 5 chloroquine/andrographolide helps in amelioration of chloroquine resistance and reduce the required dose of chloroquine to kill the parasite. Furthermore, this combination helps in shortening the period of time to achieve full eradication of the parasite.

As a multifaceted molecule, the chemo-sensitizing effect of andrographolide may be due to its impact against the multidrug resistance mechanism that aids in chloroquine accumulation within the digestive vacuoles or its impact against different biochemical pathways like the isoprenoid biosynthesis pathway. Further studies are recommended to describe the precise mechanism of its action.

## Figures and Tables

**Table 1:** Antioxidant effect of andrographolide using DPPH, hydrogen peroxide scavenging activity, or reducing power assay.

Antioxidant effect as compared with butylated hydroxytoluene		
	Andrographolide	BHT
IC_50_ hydrogen peroxide scavenging activity (*μ*g/ml)	112	32
Reducing power assay (*μ*g/ml)	46	2
DPPH assay (*μ*g/ml)	88	11

**Table 2:** *In silico* molecular characters of andrographolide.

*Physiochemical properties*	
C log *P*	1.287
*n* violation	0
MWt	350.46
nNO	5
PSA	87
nOHNH	3

*Predicted biological activity*	
GPCR ligand	0.2
Ion channel modulator	−0.08
Kinase inhibitor	−0.36
Nuclear receptor ligand	0.2
Protease inhibitor	0.13
Enzyme inhibitor	0.43

**Table 3:** Cytotoxicity of andrographolide and chloroquine against *Plasmodium falciparum*, RBCs, and Vero cells and potency classification of compounds against *Plasmodium falciparum*.

Drug IC_50_ range	Extent of potency
<1 *μ*M	Excellent potency
1 *μ*M–20 *μ*M	Good activity
20 *μ*M–100 *μ*M	Moderate activity
100–200 *μ*M	Low activity
>200 *μ*M	Inactive

**Table 4:** Cytotoxicity of andrographolide and chloroquine against *Plasmodium falciparum*, RBCs, and Vero cells and the cytotoxic effect of andrographolide against *Plasmodium falciparum* (3D7 and K1), Vero cells, and RBCs.

	Andrographolide	Chloroquine
*Part A*		
g.m.w (g/mole)	350.5	515
Molar concentration (*μ*M) equivalent to 30 *μ*g/ml	8.56	5.83

*Part B (IC*_*50*_ *values against Plasmodium falciparum 3D7 and K1, Vero cells, and RBCs)*		
IC_50_ against RBCs in *μ*M	>1000	>1000
IC_50_ against Vero cells in *μ*M	>1000	>1000
*P*. *falciparum* 3D7 *μ*M	1.4 ± 0.03	0.021 ± 0.002
SI compared with RBCs	992	>1000
SI compared with Vero cells	680	>1000
*P*. *falciparum* K1, *μ*M	1.2 ± 0.15	0.265 ± 0.05
SI compared with RBCs	965	>1000
SI compared with Vero cells	650	>1000

*Part C (IC*_*90*_ *values against Plasmodium falciparum 3D7 and K1, Vero cells and RBCs)*		
*P. falciparum* 3D7 *μ*M	23.639 ± 2.76	0.043 ± 0.001
SI compared with RBCs		
SI compared with Vero cells		
*P*. *falciparum* K1 *μ*M	28.023 ± 6.93	0.92 ± 0.05
SI compared with RBCs	280	
SI compared with Vero cells		

The table provided in part A represents the g.m.w of each item and the concentration limit in *μ*M that is equivalent to 30 *μ*g/ml; in part B, IC_50_ values against the mentioned cells as well as the IC_50_-based selectivity indices; And in subpart C, IC_90_ values against the mentioned cells as well as the IC_90_-based selectivity indices are listed.

**Table 5:** Results of the FIC_50_- and FIC_90_-based isobolograms for CQ/andrographolide mixtures.

Mixing ratio (CQ/andro)	FIC_50_-based isobologram					FIC_90_-based siobologram				
	IC_50_ CQ in nM	IC_50_ andro in *µ*M	FIC_50_ CQ	FIC_50_ andro	Total	IC_90_ CQ in nM	IC_90_ andro in *µ*M	FIC_90_ CQ	FIC_90_ andro	Total
10/0	269.2 ± 0.4	0	1.00	0.00	1.00	791.51 ± 0.00	0	1.00	0.000	1.00
7/3	184.0 ± 5.4	0.32 ± 0.02	0.68	0.30	0.99	595.58 ± 42.48	1.31 ± 0.068	0.75	0.06	0.81
5/5	128.2 ± 9.7	0.51 ± 0.05	0.48	0.49	0.97	447.20 ± 36.41	2.62 ± 0.13	0.56	0.10	0.66
3/7	95.1 ± 9.2	0.91 ± 0.03	0.35	0.85	1.21	329.83 ± 39.90	4.7 ± 0.35	0.42	0.17	0.59
0/10	0.0 ± 0.00	1.08 ± 0.00	0.00	1.00	1.00	0.00 ± 0.00	26.4 ± 0.91	0.00	1.00	1.00

## Data Availability

The figures and tables for the data used to support the findings of this study are included within the article.
